# Newly Started Versus Previously Treated Statin Patients: A Retrospective Cohort Study Comparing Adherence and Persistence with Reference to Cardiovascular Prevention

**DOI:** 10.3390/ph18050634

**Published:** 2025-04-27

**Authors:** Marta Martín-Fernández, M. Asunción González-González, M. Aránzazu Pedrosa-Naudín, Diego Fernández-Lázaro, F. Javier Álvarez, Eduardo Gutiérrez-Abejón

**Affiliations:** 1 Pharmacological Big Data Laboratory, Department of Cell Biology, Genetics, Histology and Pharmacology, Faculty of Medicine, University of Valladolid, 47003 Valladolid, Spain; marta.martin.fernandez@uva.es (M.M.-F.); alvarez@uva.es (F.J.Á.); 2 BioCritic, Group for Biomedical Research in Critical Care Medicine, 47003 Valladolid, Spain; 3 Centro de Investigación Biomédica en Red de Enfermedades Infecciosas (CIBERINFEC), Instituto de Salud Carlos III, 28029 Madrid, Spain; 4 Pharmacy Directorate, Castilla y León Health Council, 47007 Valladolid, Spain; asungonzlez@saludcastillayleon.es (M.A.G.-G.); maranzazup@saludcastillayleon.es (M.A.P.-N.); 5 Area of Histology and Neurobiology Research Group, Faculty of Medicine, University of Valladolid, 47003 Valladolid, Spain; diego.fernandez.lazaro@uva.es; 6 CEIm of Valladolid Health Areas, Hospital Clínico Universitario de Valladolid, 47003 Valladolid, Spain; 7 Atención Primaria, Área de Salud de Valladolid Este, 47010 Valladolid, Spain

**Keywords:** adherence, persistence, drug utilization, statins, inhibitors, HMG-CoA reductase, cardiovascular prevention

## Abstract

**Background/Objectives:** Cardiovascular disease (CVD) remains the leading cause of death worldwide, and the effectiveness of statin therapy is critically dependent on patient adherence and persistence. The aim of this study was to evaluate adherence and persistence in newly started and previously treated statin patients, with reference to cardiovascular prevention. **Methods:** A retrospective cohort study was conducted to assess adherence and persistence in newly started and previously treated statin patients. Patients aged 18 years or older with a statin claim from 1 January 2021 to 31 December 2023 were included. Adherence was defined as a Medication Possession Rate (MPR) of 80% or greater. Persistence was defined as the time between the index date and treatment discontinuation. Binary logistic regression and Cox proportional hazard regression were used to analyze factors influencing adherence and persistence, respectively. Kaplan–Meier survival analysis was used to compare persistence between both cohorts. **Results:** Of the 411,956 patients on statins, 81.21% were adherent, with higher rates in the previously treated statin patients (83.05% vs. 73.73%; *p* = 0.001). Statin persistence decreased from 92.65% at 3 months to 78.28% at 12 months, with higher persistence rates in previously treated statin patients. Previously treated statin patients were more likely to be adherent (AOR: 1.29) and persistent (AHR: 2.08) than those newly started on statins. In secondary prevention patients, adherence was higher in the previously treated cohort (88.09% vs. 79.77%; *p* = 0.001) than in the newly started cohort (80.52% vs. 71.38%; *p* = 0.001). Similar results were observed for persistence; 82.97% vs. 81.65% (*p* = 0.001) and 65.08% vs. 61.57% (*p* = 0.001), respectively. **Conclusions**: Adherence and persistence to statins were higher in previously treated patients than in newly started patients, especially for secondary cardiovascular prevention. New strategies are needed to improve medication adherence and persistence in patients with poor cardiovascular prognosis.

## 1. Introduction

Cardiovascular disease (CVD) is the leading cause of death worldwide. An estimated 17.9 million people died from CVDs in 2019, accounting for 32% of all global deaths [1]. Dyslipidemia is a critical predisposing factor for the development of CVD [2]. Lowering low-density lipoprotein cholesterol (LDL-C) levels with lipid-lowering therapies (LLTs) is consistently associated with a reduced risk of cardiovascular events [3]. Treatment with LLTs is a long-term, often lifelong, and risk-adjusted strategy that requires continuous monitoring to achieve and maintain target LDL-C levels [4].

The well-established benefits of statins can only be realized if patients adhere to the medication regimen [2]. Non-adherence to statin therapy remains a pervasive issue, with studies showing that a significant proportion of patients discontinue treatment within the first year, often due to side effects, cost, or lack of perceived need [5,6].

There is considerable evidence highlighting the challenges associated with adherence and persistence to statin therapy. However, a critical but understudied area in the literature is the comparison of statin use patterns between newly started and previously treated patients. Therefore, the aim of this study was to evaluate real-world medication adherence and persistence in newly started and previously treated statin patients with reference to cardiovascular prevention (primary and secondary).

## 2. Results

### 2.1. Baseline Characteristics

Between 1 January 2021 and 31 December 2023, 411,956 patients in our region used at least one statin, representing 17% of the total population. The cohort of newly started statin patients represented almost 20% of the total. Of the patients receiving statins, 51.09% were women, aged 71.10 ± 12.62 years, and nearly 70% had an annual income of less than EUR 18,000.

Most patients were receiving statin therapy for primary prevention, especially in the cohort of newly started statin patients (74.32%). Nearly 80% of patients were polymedicated and three out of four patients were on a generic statin. In addition, 5.32% of patients switched between different statins. The most commonly used statins were atorvastatin (49.59%), simvastatin (30.84%), and rosuvastatin (14.96%), with a predominance of moderate-intensity treatment (66.67%). On the other hand, almost 7% of patients were receiving concomitant treatment with other lipid-lowering drugs.

The most common comorbidities were hypertension (52.5%), anxiety (27.47%), diabetes mellitus (25.96%), and depression (20.5%). Other baseline characteristics are presented in Table 1.

### 2.2. Adherence and Persistence to Statin Treatment

Our study showed that 81.21% of the population was adherent to statin treatment, which was higher in the group of previously treated statin patients (83.05% vs. 73.73%, *p* = 0.001). Approximately 78% of the non-adherent patients had moderate MPR values between 50 and 79%. Binary logistic regression adjusted for significant variables showed that previously treated statin patients were more likely to adhere to statin therapy (AOR: 1.29; 95% CI: 1.27–1.32; *p* = 0.001) (Table 2).

**Figure 1:**
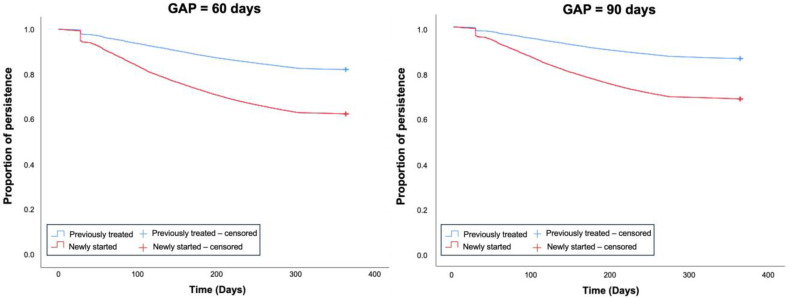
Kaplan–Meier analysis for treatment persistence in newly started and previously treated statin patients considering a 60-day gap and a 90-day gap.

Statin treatment persistence, considering a 60-day gap, was 92.65% at 3 months, decreasing to 85.56% at 6 months and 78.28% at 12 months. Persistence was significantly higher in the group of previously treated statin patients at all time intervals. The mean persistence time was 316.63 days, which was also higher in the group of previously treated statin patients (326.37 days vs. 277.15 days; *p* = 0.001). Kaplan–Meier analysis showed differences in persistence over time between patients in both cohorts. The Cox regression model adjusted for all significant variables showed that previously treated statin patients were more likely to persist on statin therapy (AHR: 2.08; 95% CI: 2.06–2.11; *p* = 0.001) (Table 2). Sensitivity analysis showed that similar results were obtained when persistence was assessed at 60 or 90 days (Figure 1).

**Figure 2:**
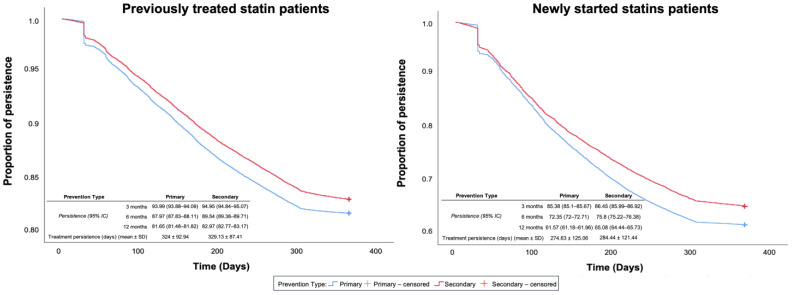
Kaplan–Meier analysis for treatment persistence in newly started and previously treated statin patients considering primary and secondary cardiovascular prevention.

Regarding the type of cardiovascular prevention, previously treated statin patients had higher adherence and prevalence in secondary prevention: 88.09% vs. 79.77% (*p* = 0.001) and 82.97% vs. 81.65% (*p* = 0.001), respectively. The same behavior was observed in newly started statin patients: 80.52% vs. 71.38% (*p* = 0.001) and 65.08% vs. 61.57% (*p* = 0.001), respectively (Figure 2).

## 3. Discussion

Our analysis of real-world adherence and persistence to statin therapy among patients in Castilla y León, Spain, shows that both adherence and persistence were significantly higher among previously treated statin patients. These findings indicate that prior treatment with statin therapy is associated with improved adherence and persistence, suggesting that familiarity with statin use may positively influence adherence behavior. Our findings are consistent with those reported for other cardiovascular medicines such as oral anticoagulants [7].

To our knowledge, this is the first study to compare adherence and persistence patterns between newly started and previously treated statin patients, highlighting the potential benefit of prior therapy in supporting sustained statin use. There are currently no studies in the literature that measure adherence and persistence between newly started patients and previously treated statin patients, so comparisons with our study are limited. However, the interpretation of this behavior could be extrapolated from studies with other cardiovascular medicines. In this sense, the prior treatment of patients increases their awareness of the negative consequences of non-adherence. In addition, prior experience has been observed to positively influence individuals to take medications according to the prescribed regimen [7]. Undoubtedly, these attitudes improve adherence and persistence to statin therapy.

Our study showed that the prevalence of statin adherence in the population of the largest region of Spain was 81.21%. These results are consistent with those of other studies, such as that of Koenig et al. [4], which reported statin adherence of 84%. However, other studies show lower adherence rates compared to our results [8,9]. Regarding the persistence of statin treatment, our study showed a persistence of 78.28% at 12 months. These results are slightly higher than those reported in other studies, where one-year persistence with statins ranges from 40% to 70% [4,8,9,10,11,12].

As in other studies [11,13,14,15], our results show that secondary prevention patients are more adherent and persistent on statins than primary prevention patients. Improved therapeutic compliance in patients with cardiovascular disease may be associated with a greater perception of the importance of risk control and the expected benefits [13,16].

Patterns of statin prescribing by intensity can be confusing. Several studies [11,17] show that treatment with high-intensity statins predominates. However, as in other studies [8,18], patients on moderate-intensity statin therapy predominated in our region. This behavior may be explained by the aging of the population and the predominant use of statins for primary prevention [19,20]. This discrepancy in results between studies highlights the difference between the recommendations of clinical practice guidelines [21] and standard clinical practice. Unfortunately, we cannot justify the efficacy of statin therapy in our patients due to the lack of LDL-C levels.

The concomitant use of other lipid-lowering drugs was similar to that in another Spanish study [18]. The likelihood of discontinuation of treatment with another lipid-lowering drug has been shown to be higher than when a moderate-intensity statin is prescribed [22]. However, combination with other lipid-lowering drugs in newly started statin patients [23] is associated with a lower likelihood of discontinuation [8]. In addition, the efficacy of adding ezetimibe or PCKSK9 inhibitors to statin therapy in reducing LDL-C levels has been widely demonstrated [24,25].

The clinical significance of our findings is considerable. There is increasing evidence that poor adherence to statins and early discontinuation are associated with worse clinical outcomes [26]. For example, patients who discontinue statin therapy after myocardial infarction (MI) are almost three times more likely to die than those who continue treatment [14]. In addition, patients who are non-adherent to both statins and antihypertensive medications have a more than sevenfold increased risk of fatal stroke compared with adherent patients [27]. Not surprisingly, a meta-analysis reported 9 additional cardiovascular deaths per 100,000 people due to non-adherence to cardiovascular medications, including statins [26]. These challenges would be exacerbated in primary prevention patients, who already have lower adherence and persistence rates, with an even more pronounced decline observed in naïve patients.

Definitively, lower adherence and persistence in newly started statin patients impairs the achievement and maintenance of LDL-C targets in newly diagnosed patients, increasing cardiovascular morbidity and mortality [28,29]. This behavior should serve as a starting point for implementing strategies to educate these patients, especially those who are taking statins for secondary prevention.

Socioeconomic level was considered important to include in the adjustment of binary logistic regression and Cox proportional hazard regression. The Spanish healthcare system is universal, but patients must pay out-of-pocket for medicines. In Spain, statins are subject to a co-payment that is stratified according to the patient’s socioeconomic level, ranging from 0% for the lowest income to 60% for the highest [30]. In this sense, González López-Valcarcel et al. [31] previously observed reductions of up to about 8% in adherence to statins and other cardiovascular medications.

Patients on statin therapy have a high rate of polypharmacy, especially in previously treated patients. Other studies previously conducted in Spain found that adherence to psychotropic medications was up to 10% higher in polymedicated patients than in non-polymedicated patients [32,33,34]. These improved results may be explained by the high percentage of comorbidities in polymedicated patients, who require more intensive physician follow-up [35]. In addition, risk perception is higher in complex patients and is associated with higher levels of adherence and persistence [13,16].

Our study has limitations that should be mentioned. We assumed that dispensing approximated consumption, which may have underestimated the prevalence of adherence and persistence. Furthermore, we lack data on statin use in hospitals and on private prescriptions, as this information is not available in our data source, CONCYLIA. However, as the sample size is large and the statins covered are prescription drugs, the biases introduced by dispensing data are not considered relevant. On the other hand, no data are available on the rate of statin-associated side effects in our cohort. This can be a handicap, as side effects such as muscle-related events or rhabdomyolysis are associated with lack of adherence to LLTs [36], especially in women [37,38] and polymedicated patients [39]. Another limitation of our study is the possible misclassification of some patients as primary prevention. This is because the type of prevention was determined by the medication used for coronary heart disease before the index date. This limitation is common to other similar published studies [10,13]. Finally, no data are available on the potential impact on adherence and persistence of myocardial infarction in previously treated statin patients, or whether newly started patients become previously treated patients during follow-up. Prospective studies with smaller cohorts could be conducted in the future to further investigate these issues.

## 4. Materials and Methods

### 4.1. Study Design and Data Source

We conducted an epidemiologic, population-based registry cohort study, following the recommendations of Strengthening the Reporting of Observational Studies in Epidemiology (STROBE) [40] and the REporting of studies Conducted using Observational Routinely collected Health Data for Pharmacoepidemiology (RECORD-PE) [41]. This study was conducted in Castilla y León, a region of Spain with a population of 2,390,321, and focused on patients receiving statin treatment in 2021–2023. Claims data were obtained from the Pharmaceutical Information System of Castilla y León (CONCYLIA) [42], which contains primary care prescribing and dispensing data for all patients covered by the Spanish National Health Service, approximately 97% of the population.

Informed consent was not required because CONCYLIA anonymizes patient data. The Ethics Committee of the Valladolid Health Areas approved this study on 27 July 2023 (reference number PI-GR-3265 AP).

### 4.2. Study Population

**Figure 3:**
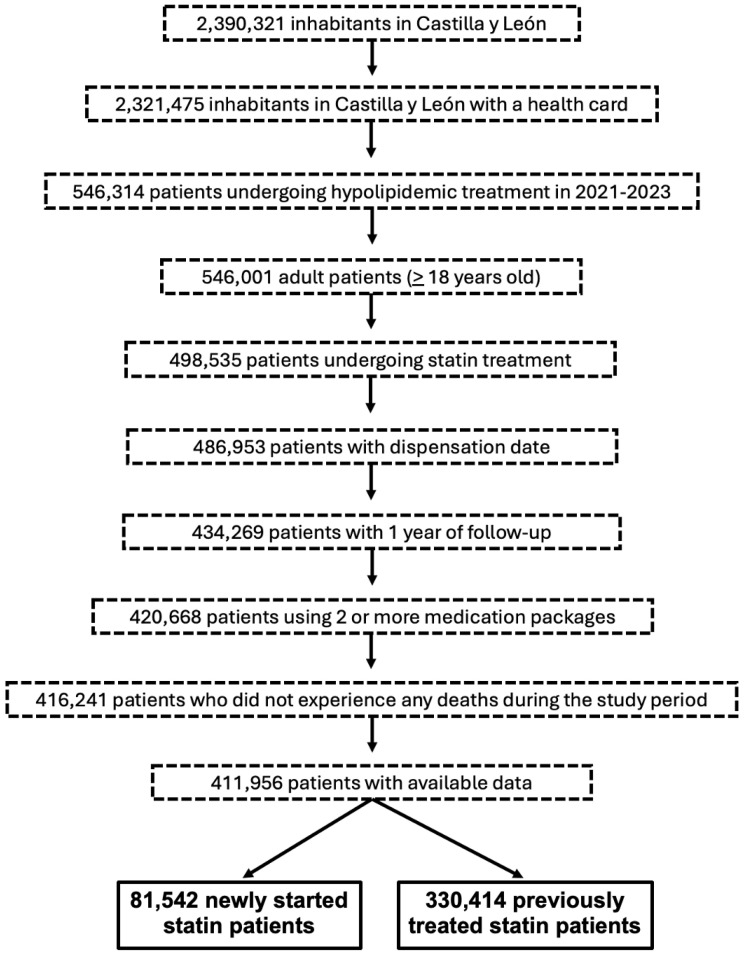
Flowchart of the patient selection process for the study.

Finally, a total of 411,956 patients on statin therapy were selected from the entire adult population of Castilla y León and divided into two cohorts: newly started and previously treated statin patients (Figure 3). Patients were classified as new users if they had not received a statin in the 12 months prior to the index date.

Patients who died during the study period, had inconsistent medication records (date of dispensing not available), or had a follow-up of less than 12 months were excluded.

### 4.3. Study Variables and Definitions

Sociodemographic data included sex, age, institutionalization, healthcare area, and socioeconomic level. Clinical data included type of prevention; statin type (stratified by intensity of treatment); treatment with other lipid-lowering drugs; concomitant medication or polypharmacy; generic treatment; switching; multiple prescribers and multiple pharmacies (≥3/year); comorbidities; adherence; persistence at 3, 6, and 12 months; and mean persistence (in days).

Secondary prevention of cardiovascular disease was defined as the use of medications for coronary artery disease (ATC [43] code B01 “antithrombotic agents” and C01 “cardiac therapy”) before the index date [13]. Intensity of statin therapy was defined as follows: low intensity (20–40 mg fluvastatin, 10–20 mg lovastatin, 1–4 mg pitavastatin, 5–10 mg simvastatin, or 10–20 mg pravastatin per day), moderate intensity (10–30 mg atorvastatin, 80 mg fluvastatin, 40 mg lovastatin, 20–40 mg simvastatin, 30–40 mg pravastatin, or 5–10 mg rosuvastatin per day), and high intensity (≥40 mg atorvastatin, 80 mg simvastatin, or ≥20 mg rosuvastatin per day) [4,22]. A polymedicated patient was defined as a patient using 5 or more medications as established by the World Health Organization [44].

The Medication Possession Rate (MPR) was used as a measure of adherence, calculated as the number of days’ supply during a specified follow-up period (365 days) divided by the number of days from first dispensing to the end of the follow-up period [45]. According to the MPR value, adherence was classified as none (<20), poor (20–49), moderate (50–79), and adherent (≥80) [32,33,46].

Persistence was defined as the time between the index date and discontinuation. A gap of >60 days between prescription refills for any statin was considered discontinuation. The percentage of persistent patients at 3, 6, and 12 months was calculated. Statin switching (different ATC code) was considered as discontinuation. Sensitivity analyses were performed by changing the allowed gap between prescription refills from 60 to 90 days.

### 4.4. Statistical Analysis

Prevalences or percentages with their 95% confidence intervals (95% CI) or means with their standard deviation (SD) were used to report descriptive statistics, as appropriate. Kolmogorov–Smirnov and Shapiro–Wilk tests were used to assess the normality of distributions. Differences between continuous and categorical variables were analyzed using Student’s t-test and Pearson’s chi-squared test (χ^2^), respectively.

Factors influencing adherence were analyzed by binary logistic regression, with results expressed as adjusted odds ratios (AORs) with 95% CIs. Persistence and duration of treatment in both cohorts were analyzed by Kaplan–Meier survival analysis using the log-rank test. Absence of an event (discontinuation) resulted in censoring of the data. Persistence at 1 year was analyzed by Cox proportional hazard regression. Binary logistic regression and Cox proportional hazard regression were adjusted for covariates: sex, age, healthcare area, socioeconomic level, type of prevention, polypharmacy, switching, statin, and treatment intensity. All study variables were included in the univariate analysis, whereas only variables with *p* ≤ 0.05 in the univariate analysis were included in the multivariate analysis.

SPSS version 29.0 (SPSS Inc., Chicago, IL, USA) was used for statistical analysis. Statistical significance was set at *p* ≤ 0.05.

## 5. Conclusions

In conclusion, this study is one of the first to shed light on the differences in adherence and persistence between newly started and previously treated statin patients. In this retrospective cohort study, adherence and persistence were higher in previously treated than in newly started statin patients, especially in secondary cardiovascular prevention.

One of the most important contributions of our study is the development of robust indicators to identify non-adherent and non-persistent patients on statins. These indicators have been implemented in the regional public health system [32,33] to identify non-compliant patients so that healthcare professionals can reinforce the message of proper medication use [47]. Another important use of these indicators is to identify subpopulations at higher risk for the consequences of non-adherence and persistence, which is particularly relevant for patients at cardiovascular risk [48].

Finally, new strategies to improve medication adherence and persistence in patients with poor cardiovascular prognosis are needed to optimize clinical management and effectively monitor cardiovascular risk.

## Figures and Tables

**Table 1:** Baseline characteristics.

	Total	Previously Treated Statin Patients	Newly Started Statin Patients
***n***	411,956	330,414	81,542
**Sociodemographic characteristics, *n* (%)**			
*Sex*			
Female	210,468 (51.09)	166,760 (50.47)	43,731 (53.63)
*Age (mean ± SD)*	71.10 ± 12.62	72.73 ± 12.03	64.51 ± 12.82
*Distribution by age groups*			
*18–44*	8280 (2.01)	3899 (1.18)	4371 (5.36)
*45–64*	117,737 (28.58)	79,960 (24.2)	37,770 (46.32)
*65–74*	116,542 (28.29)	95,159 (28.8)	21,356 (26.19)
*≥75*	169,438 (41.13)	151,396 (45.82)	18,045 (22.13)
*Institutionalized*	11,947 (2.9)	9979 (3.02)	1933 (2.37)
*Healthcare area*			
*Rural*	197,615 (47.97)	158,797 (48.06)	38,838 (47.63)
*Urban*	214,341 (52.03)	171,617 (51.94)	42,704 (52.37)
*Socioeconomic level*			
*<18,000 EUR/year*	275,681 (66.92)	222,798 (67.43)	52,904 (64.88)
*18,000–100,000 EUR/year*	133,680 (32.45)	105,666 (31.98)	28,002 (34.34)
*>100,000 EUR/year*	2595 (0.63)	1949 (0.59)	636 (0.78)
**Clinical characteristics, *n* (%)**			
*Prevention type*			
*Primary*	259,244 (62.93)	198,645 (60.12)	60,594 (74.31)
*Secondary*	152,712 (37.07)	131,769 (39.88)	20,948 (25.69)
*Polypharmacy*	319,513 (77.56)	260,961 (78.98)	58,555 (71.81)
*Polypharmacy level (nº drugs)*			
*5–10*	193,784 (47.04)	156,583 (47.39)	37,191 (45.61)
*11–15*	84,739 (20.57)	70,477 (21.33)	14,237 (17.46)
*>15*	41,031 (9.96)	33,900 (10.26)	7127 (8.74)
*Generic statin treatment*	318,978 (77.43)	253,461 (76.71)	65,519 (80.35)
*Multiple prescribers*	194,690 (47.26)	155,361 (47.02)	39,344 (48.25)
*Multiple pharmacies*	138,664 (33.66)	109,697 (33.2)	28,956 (35.51)
*Switching*	21,916 (5.32)	15,529 (4.7)	6401 (7.85)
**Statin use, *n* (%)**			
*Drug*			
*Atorvastatin*	204,289 (49.59)	162,167 (49.08)	42,100 (51.63)
*Simvastatin*	127,047 (30.84)	105,600 (31.96)	21,421 (26.27)
*Rosuvastatin*	61,629 (14.96)	43,053 (13.03)	18,583 (22.79)
*Pravastatin*	14,666 (3.56)	13,349 (4.04)	1329 (1.63)
*Pitavastatin*	13,924 (3.38)	11,333 (3.43)	2593 (3.18)
*Fluvastatin*	4614 (1.12)	4461 (1.35)	155 (0.19)
*Lovastatin*	1689 (0.41)	1619 (0.49)	90 (0.11)
*Treatment intensity*			
*Low Intensity*	75,882 (18.42)	63,506 (19.22)	12,378 (15.18)
*Moderate Intensity*	274,651 (66.67)	215,826 (65.32)	58,833 (72.15)
*High Intensity*	80,620 (19.57)	64,596 (19.55)	16,007 (19.63)
*Other lipid-lowering drugs*	27,601 (6.7)	23,294 (7.05)	4322 (5.3)
**Comorbidities, *n* (%)**			
*Hypertension*	216,277 (52.5)	183,281 (55.47)	32,984 (40.45)
*Anxiety*	113,164 (27.47)	92,318 (27.94)	20,858 (25.58)
*Diabetes Mellitus*	106,944 (25.96)	91,393 (27.66)	15,575 (19.1)
*Depression*	84,451 (20.5)	68,759 (20.81)	15,697 (19.25)
*Ischaemic heart disease*	76,541 (18.58)	66,215 (20.04)	10,331 (12.67)
*Heart failure*	62,864 (15.26)	53,857 (16.3)	8986 (11.02)
*Respiratory disease*	55,532 (13.48)	44,044 (13.33)	11,489 (14.09)
*Thyroid disease*	49,888 (12.11)	40,476 (12.25)	9418 (11.55)
*Gout*	38,559 (9.36)	33,306 (10.08)	5268 (6.46)
*Psychotic illness*	28,425 (6.9)	23,294 (7.05)	5145 (6.31)
*Osteoporosis*	14,336 (3.48)	12,060 (3.65)	2259 (2.77)
*Dementia*	6550 (1.59)	5815 (1.76)	734 (0.9)

*Abbreviations: SD, standard deviation.*

**Table 2:** Evaluation of differences in adherence to and persistence of statin therapy between newly started and previously treated statin patients.

	Total	Previously Treated Statin Patients	Newly Started Statin Patients	*p*
***n***	411,956	330,414	81,542	
Statin Adherence Prevalence				
*MPR (mean ± SD)*	88.63 ± 15.82	89.59 ± 14.70	84.78 ± 19.24	0.001
*Adherence prevalence (95% CI)*	81.21 (81.09–81.33)	83.05 (82.92–83.18)	73.73 (73.43–74.03)	0.001
*Adherence level (95% CI)*				
None (<20)	0.56 (0.54–0.58)	0.3 (0.28–0.32)	1.62 (1.54–1.71)	0.001
Poor (20–49)	3.65 (3.59–3.71)	3.13 (3.07–3.19)	5.75 (5.59–5.91)	
Moderate (50–79)	14.58 (14.47–14.69)	13.51 (13.4–13.63)	18.89 (18.63–19.16)	
Logistic regresion model *				
OR (95% CI)		1.75 (1.72–1.78)		0.001
AOR (95% CI)		1.29 (1.27–1.32)		0.001
Treatment persistence (GAP = 60 days)				
*Persistence (95% IC)*				
3 months	92.65 (92.57–92.73)	94.37 (94.29–94.45)	85.66 (85.42–85.9)	0.001
6 months	85.56 (85.45–85.66)	88.6 (88.49–88.71)	73.24 (72.93–73.54)	0.001
12 months	78.28 (78.15–78.4)	82.18 (82.04–82.31)	62.47 (62.14–62.8)	0.001
*Treatment persistence (days) (mean ± SD)*	316.63 ± 100.26	326.37 ± 90.80	277.15 ± 124.21	0.001
Cox proportional hazards model *				
HR (95% CI)		2.42 (2.39–2.46)		
AHR (95% CI)		2.08 (2.06–2.11)		

* Reference group for binary regression model and Cox proportional hazards model: previously treated statin patients. Abbreviations: SD, standard deviation; 95% CI, confidence interval; OR, odds ratio; AOR, adjusted odds ratio; HR, hazard ratio; AHR, adjusted hazard ratio.

## Data Availability

Restrictions apply to the availability of these data. Data were obtained from regional health authorities (Gerencia Regional de Salud (GRS)) and may be requested from conciertofco@saludcastillayleon.es (GRS).
